# Molecular Magnetic Resonance Imaging of Tumor Response to Therapy

**DOI:** 10.1038/srep14759

**Published:** 2015-10-06

**Authors:** Adam J. Shuhendler, Deju Ye, Kimberly D. Brewer, Magdalena Bazalova-Carter, Kyung-Hyun Lee, Paul Kempen, K. Dane Wittrup, Edward E. Graves, Brian Rutt, Jianghong Rao

**Affiliations:** 1 Molecular Imaging Program at Stanford, Stanford, California 94305, USA; 2 Departments of Radiology, Stanford, California 94305, USA; 3 Radiation Oncology, Stanford, California 94305, USA; 4 Materials Science and Engineering, Stanford University, Stanford, California 94305, USA; 5 Department of Chemical Engineering, Department of Biological Engineering, and Koch Institute for Integrative Cancer Research, Massachusetts Institute of Technology, Cambridge, Massachusetts, 02139, USA

## Abstract

Personalized cancer medicine requires measurement of therapeutic efficacy as early as possible, which is optimally achieved by three-dimensional imaging given the heterogeneity of cancer. Magnetic resonance imaging (MRI) can obtain images of both anatomy and cellular responses, if acquired with a molecular imaging contrast agent. The poor sensitivity of MRI has limited the development of activatable molecular MR contrast agents. To overcome this limitation of molecular MRI, a novel implementation of our caspase-3-sensitive nanoaggregation MRI (C-SNAM) contrast agent is reported. C-SNAM is triggered to self-assemble into nanoparticles in apoptotic tumor cells, and effectively amplifies molecular level changes through nanoaggregation, enhancing tissue retention and spin-lattice relaxivity. At one-tenth the current clinical dose of contrast agent, and following a single imaging session, C-SNAM MRI accurately measured the response of tumors to either metronomic chemotherapy or radiation therapy, where the degree of signal enhancement is prognostic of long-term therapeutic efficacy. Importantly, C-SNAM is inert to immune activation, permitting radiation therapy monitoring.

Current clinical assessment of tumor response to radiation or chemotherapy relies upon volumetric measurements and morphological criteria obtained from magnetic resonance imaging (MRI) or x-ray computed tomography (CT)^1^,^2^,^3^,^4^. These techniques utilize serial bidimensional or unidimensional ellipsoidal approximations of tumors, and compare observed changes with response thresholds defined by the World Health Organization (WHO)^2^,^5^, or Response Evaluation Criteria in Solid Tumors (RECIST)^6^,^7^, respectively. The limitations of such techniques for monitoring tumor therapy response derive from both their dependence on tumor anatomical changes as well as their susceptibility to inter-observer variability due to lesion irregularity^4^,^8^, and the inability to assess efficacy early (within days) after treatment^2^. This inability to reliably measure early therapy response in the clinic can result in prolonged periods of inappropriate therapy that limit treatment efficacy and cancer survivability, and significantly increase the cost of care^1^,^3^. However, these barriers to treatment monitoring can be overcome by utilizing molecular-level diagnostic data to rationally select individualized approaches to anti-cancer therapy^1^,^3^.

Molecular changes to tumor tissue following treatment precede changes in tumor morphology^2^,^9^,^10^, and include key events driving therapy-induced tumor cell death. Current methods providing molecular-level information of tumor response to therapy involve biopsy-based tissue sampling of discrete tumor regions, which, in addition to being invasive, delayed, and impractical for serial observation, inadequately predict tumor response due to the characteristic heterogeneity of tumor tissue^2^,^11^. Alternatively, ^18^F-fluorodeoxyglucose (FDG) positron emission tomography (PET) has been used to assess therapeutic response non-invasively over the entire tumor volume: a positive response is indicated by a reduction in standardized uptake value (SUV) over the entire tumor region of interest^10^,^12^. However, this imaging method resulting in a reduction of tumor signal requires comparisons to pre-treatment imaging^8^, and is limited in its utility when applied to therapies that induce FDG-avid inflammation such as radiation therapy^10^,^12^. These limitations demand new clinical molecular imaging strategies in order to more robustly monitor the response of tumors to both radiation and chemotherapy.

MRI is an alternative modality to PET with higher spatial resolution and the ability to simultaneously acquire anatomical and molecular-level, contrast agent-dependent images in the same scan, free from ionizing radiation that could cause secondary cancer^2^,^9^. However, MRI suffers from low detection sensitivity that impedes the successful design of molecular MRI contrast agents that can image biological processes at the cellular and subcellular level. We have recently described a small molecule imaging probe scaffold unique in its ability to undergo self-assembly into nanoparticles in living animals when acted upon by a target enzyme of interest^13^,^14^,^15^. This probe scaffold provides three signal amplification mechanisms that we hypothesize will overcome the low sensitivity associated with MRI, and facilitate molecular MR imaging. Firstly, the probe is a substrate for its enzyme target, affording many probe activation events per active target biomolecule^14^,^15^. Secondly, nanoparticles exhibit prolonged tissue retention, producing localized regions of signal enhancement in the direct microenvironment of the activated target enzyme while unactivated probe is washed out from surrounding tissue^13^,^14^,^15^. Thirdly, and unique to MRI, the increase in contrast agent size from small molecule to nanoparticle enhances the relaxivity of the self-assembled product^14^, and directly impacts signal generation^16^.

Herein we have applied this probe scaffold to design an MRI substrate probe for caspases 3 and 7, effector cysteine-aspartate proteases that commit the cell to die, with caspase-3 being critically involved in both chemotherapy and radiation therapy-induced tumor eradication^17^,^18^. This work represents an in depth investigation of the ability of our caspase-sensitive nanoaggregation MRI contrast agent (C-SNAM) to monitor therapy response in animal models of tumor therapy that approximate metronomic doxorubicin chemotherapy or x-ray beam radiation therapy relative to non-aggregatable control probes. The implementation of C-SNAM was developed by assessing the timing of maximal imaging response after treatment for the first time, as this is a current clinical challenge in molecular imaging of dynamic biological targets reporting on therapeutic response^18^. Additionally, the correlation of contrast agent activation with treatment efficacy was defined in this work, which indicated that a single post-treatment imaging session was able to effectively assess therapeutic efficacy. Utilizing our strategy of enzyme-triggered self-assembly *in vivo*, which integrates three mechanisms of signal amplification, one of which is unique to MRI, monitoring of treatment response for both chemotherapy and radiation therapy by *in vivo* imaging is shown to be possible.

## Results

### Mechanism of C-SNAM Activation and Self-Assembly

**Figure 1:**
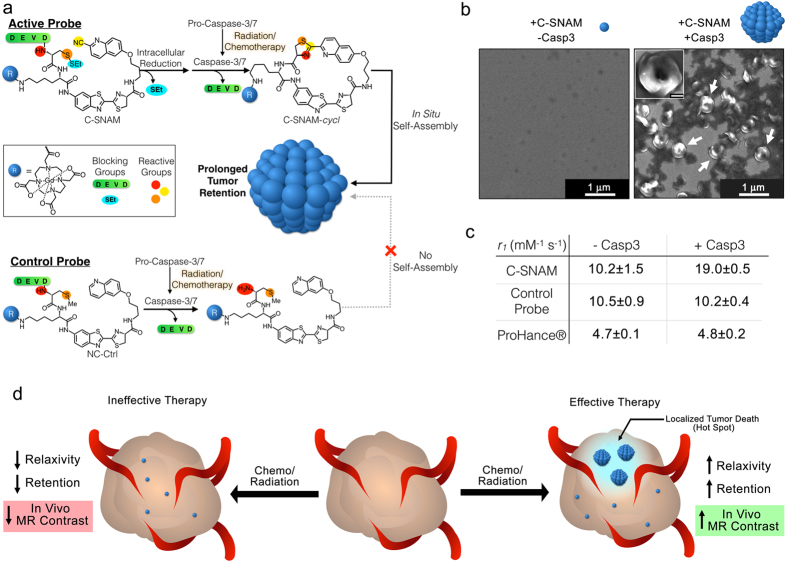
Structure, mechanism of action, and *in vitro* characterization of caspase 3-sensitive nano-aggregation MRI contrast agent (C-SNAM). (**a**) Schematic showing the structure of C-SNAM, including the DOTA-chelated Gd (blue sphere), the DEVD (green) and thioethyl (SEt, cyan) blocking groups, and the amine (red), thiol (orange), and cyano (yellow) click chemistry reactive groups. Upon removal of the capping groups, C-SNAM cyclizes and self-assembles into nanoparticles. The structure of the non-cyclizable control probe (NC-ctrl) lacks the cyano group and contains a methylated thiol, preventing cyclization upon removal of DEVD. (**b**) Scanning electron micrographs of C-SNAM alone (left) and after incubation with activated caspase-3 (right), showing self-assembled nano-aggregates (white arrows). (**c**) The activation of C-SNAM by caspase-3 results in an increase in relaxivity (*r*_1_) at 1T, but neither the activation of control probe (NC-ctrl) nor ProHance ®, suggesting that the change in *r*_i_ is a result of the increase in contrast agent size following nano-aggregation. (**d**) Proposed mechanism of therapy response monitoring using C-SNAM. With ineffective therapy, caspase-3 remains inactive, failing to trigger C-SNAM self-assembly and resulting in rapid clearance of the probe. With successful tumor cell death, caspase-3 is activated and induces C-SNAM self-assembly, increasing signal retention and enhancing signal production to result in localized regions of MR signal enhancement (i.e. hotspots).

C-SNAM is composed of a flexible aminoluciferin-based linker connecting two biocompatible reactive moieties, D-cysteine on one end (Fig. 1a, red and orange) and 2-cyano-6-hydroxyquinoline (Fig. 1a, yellow) on the other. In order to control the bioorthogonal reaction and self-assembly, the reactive groups of D-cysteine are reversibly blocked: the thiol is blocked by a disulfide bond to a thioethyl group (cyan oval) reducible by glutathione upon entry into a cell. The amine is blocked by an acetyl-Asp-Glu-Val-Asp (DEVD, green bar) group, a substrate of caspase-3 that is removed upon activation of the enzyme during successful radiation or chemotherapy^17^,^18^. When unblocked, these reactive groups undergo rapid intramolecular condensation under physiological conditions (first order rate constant = 5.8 × 10^−3^ s^−1^)^19^ to form a cyclic compound, C-SNAM-*cycl*, whose high rigidity favors self-assembly through π-π stacking, ultimately forming a nanoparticle at a typical size of approximately 400 nm (Fig. 1b)^14^,^15^,^19^. In addition to C-SNAM, a non-cyclizable control probe (NC-ctrl) was prepared lacking the cyano moiety and having a methylated thiol, preventing cyclization even after removal of the DEVD blocking group to abrogate self-assembly (Fig. 1a).

C-SNAM contains a DOTA-Gd^3+^ conjugate for MRI signal enhancement through water proton relaxation. Upon activation, the spin-lattice relaxivity (r_1_) substantially increased from 10.2 ± 1.5 mM^−1^ s^−1^ in the absence of caspase-3 to 19.0 ± 0.5 mM^−1^ s^−1^ in the presence of caspase-3 at 1T (Fig. 1c). Neither the control probe nor the clinically used T_1_-contrast agent ProHance® showed any substantial change in r_1_ between incubation conditions (Fig. 1c). The building of nanoparticles *in situ* induces a substantial increase in the size of the product contrast agent nanoaggregate relative to its small molecule precursor, as we have directly observed through superresolution microscopy in tissue samples *ex vivo* 14,15. Since an increase in size of the contrast agent increases the rotational correlation time constant (τ_R_), which is a dominant influence that directly correlates with r_1_ of the contrast agent at the high frequencies employed by medical imaging, an increase in τ_R_ can result in a substantial increase in signal enhancement^16^. With this signal amplification strategy, it is hypothesized that following ineffective chemo- or radiation therapy and therefore a lack of caspase-3 activation, C-SNAM will not be activated but remain as a small molecule with low relaxivity and be rapidly cleared from the tumor (Fig. 1d, left). However, with effective therapy inducing localized regions of tumor death and caspase-3 activation, C-SNAM will be activated to self-assemble. Within these localized regions, or hotspots, the combination of enhanced relaxivity of the resulting nanoparticle with prolonged tumor retention will result in substantially elevated MRI signal enhancement to signal tumor cell death response to treatment (Fig. 1d, right).

### Validation of Animal Models of Cancer Chemotherapy & Radiation Therapy

**Figure 2:**
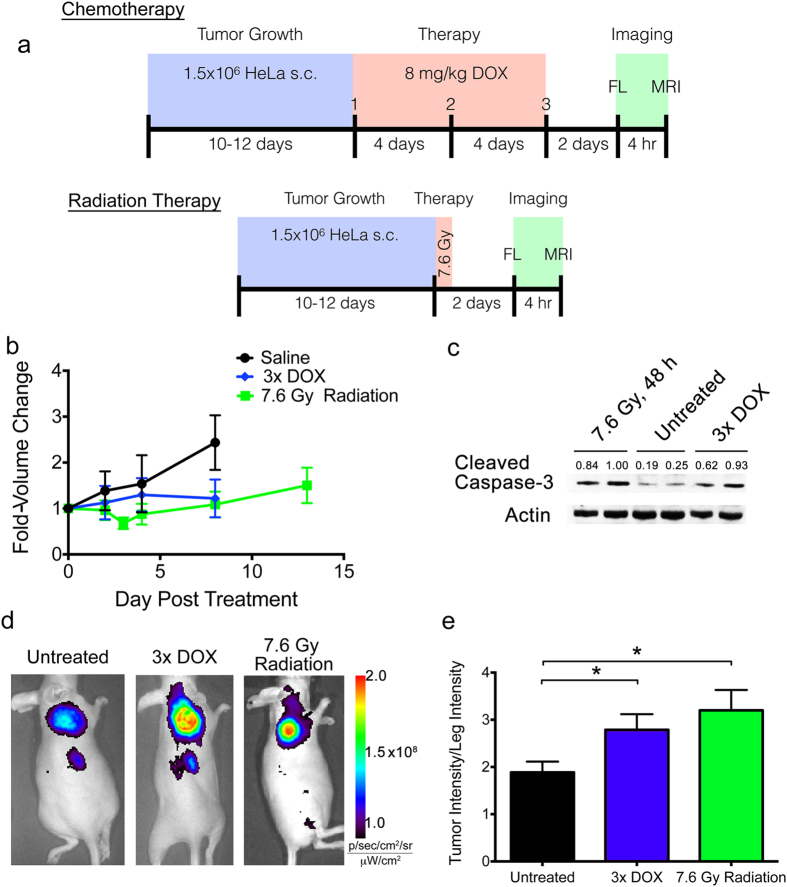
Animal models of metronomic chemotherapy and radiation therapy. (**a**) The scheme for generating each animal model is provided, where blue represents tumor growth phase, red represents treatment phase, and green represents imaging phase. (**b**) Tumor size was measured over time from the initiation of treatment, with fold-volume change relative to pre-treatment provided for untreated (black), metronomic chemotherapy (blue, 3x DOX) or radiation treatment (green, 7.6 Gy Radiation). (**c**) Western blot showing activation of caspase-3 (cleaved caspase-3) two days following radiation therapy or the end of metronomic chemotherapy. Values are cleaved caspase-3 band intensities normalized to actin loading control. (**d**) Prior to MR imaging, caspase-3 activation in tumors was determined using our fluorescent, quenched C-SNAM analog (Q-C-SNAF), confirming elevated caspase-3 activity following radiation and chemotherapy relative to untreated animals. (**e**) Tumor-to-leg fluorescence intensity ratio is provided for untreated (black), or mice treated with metronomic chemotherapy (blue) or radiation therapy (green). *p < 0.05 (ANOVA), n = 4.

HeLa tumor-bearing nude mice were left either untreated, or received doxorubicin chemotherapy (metronomic, 8 mg/kg q4d)^15^ or single dose radiation therapy (7.6 Gy with 225 kV beam) through a lead shield exposing only the tumor to radiation, with imaging performed 2 days after the final treatment (Fig. 2a). Doxorubicin dose was selected to approximate clinical metronomic chemotherapeutic doses^20^, and the radiation treatment mimics a single fractionated dose received during current stereotactic ablative radiotherapy treatment regimens employed in the clinic^21^. Both the metronomic chemotherapy and the radiation therapy resulted in a substantial tumor growth delay relative to the untreated controls (Fig. 2b), but did not result in a reduction of tumor size relative to pre-treatment. However, the therapy-induced tumor growth delay was accompanied by an elevation of caspase-3 activation in both treatment groups (Fig. 2c). *In vivo* caspase-3 activity was assayed 4 hr prior to MRI utilizing a quenched fluorescent caspase-3 probe, Q-C-SNAF, we have previously reported^15^ (Supplementary Fig. S1). This imaging result confirmed more extensive fluorescence retention in chemo- or radiation-treated tumors (Fig. 2d) and a significant enhancement of tumor caspase-3 activity normalized to background intensities quantified in the leg (ANOVA, *P* < 0.05, Fig. 2e). These results demonstrate that the chosen clinically relevant metronomic chemotherapy and low-dose radiation therapy models selected provide elevated levels of caspase-3, and support their suitability for the interrogation of therapy response monitoring by C-SNAM through molecular MRI.

### Chemotherapy Response Monitoring with C-SNAM

**Figure 3:**
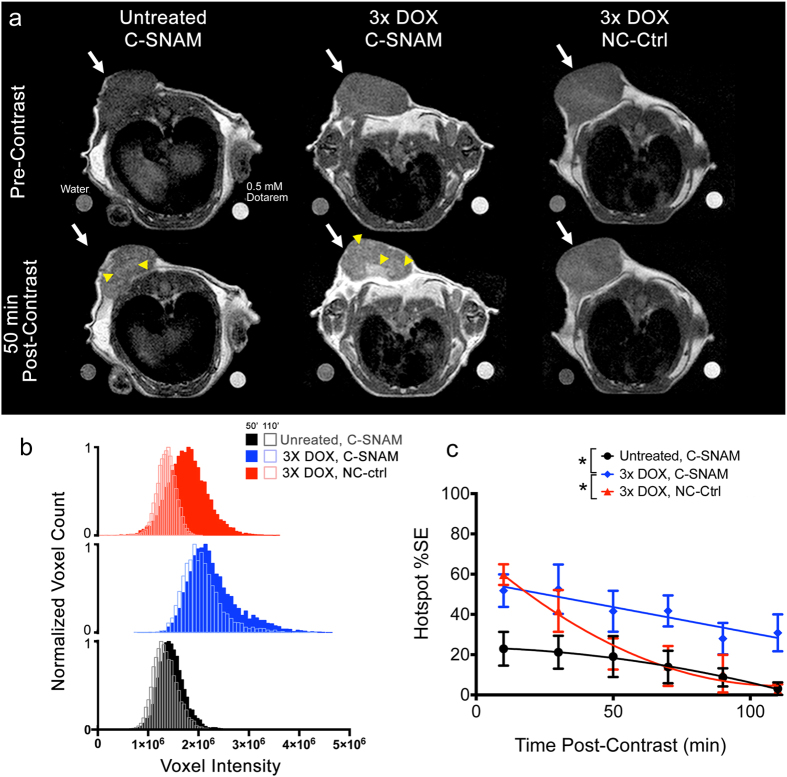
Imaging the therapeutic response to metronomic chemotherapy in HeLa tumor-bearing mice. (**a**) Representative MR images of untreated (left) and treated mice (center) receiving C-SNAM, and treated mice receiving NC-ctrl (right) pre-contrast (top) and 50 min post-contrast (bottom). Tumor is indicated by white arrow, hot spot is indicated by yellow arrowhead. Water and 0.5 mM Dotarem phantoms are shown on bottom left and right, respectively, of each image. (**b**) Representative voxel histograms for untreated (black) and treated (blue) mice receiving C-SNAM, and treated mice receiving NC-ctrl (red). Histograms are shown 50 min post-contrast (solid bars) and 110 min post-contrast (open bars). (**c**) The percent signal enhancement (%SE) for the hotspot signal enhancement (mean signal within the top quartile of the enhancement histogram) is plotted over time for untreated (black) and treated (blue) mice receiving C-SNAM, and treated mice receiving NC-ctrl (red). Values are mean ± s.d., *p < 0.05 (general linear model repeated measures), and n = 4 per group.

A single MRI imaging session at 1T was conducted 2 days following the final dose of doxorubicin chemotherapy, identified as the peak of caspase-3 activity based on previous study with this model^15^. Images were acquired prior to C-SNAM injection (pre-contrast) and every 10 min following C-SNAM injection (post-contrast). Reference standards containing water (left of the mouse) or 0.5 mM Dotarem® (right of the mouse) were imaged to allow for the normalization of image contrast as previously described^22^,^23^. Pre- and post-contrast images were used to normalize between scan sessions. Representative images of a tumor pre-contrast (Fig. 3a, top row) and 50 min post-contrast (Fig. 3a, bottom row) are provided for untreated (left) and mice treated with metronomic chemotherapy and administered C-SNAM (middle), and treated mice administered the non-cyclizable NC-ctrl probe (right). Hotspots of C-SNAM activation and retention can be observed in the treated mice receiving active probe (Fig. 3a, yellow arrowheads), with fewer and less intense hotspots notable in untreated mice, and in treated mice receiving NC-ctrl control probe. While tumor-averaged signal enhancement was not substantially different between the three groups (Supplementary Fig. S2a), significant differences were revealed through analysis of hotspot signal (General linear model repeated measure analysis, P < 0.05). In order to analyze the appearance of hotspots, tumor signals were quantified through voxel signal histograms over the entire volume of the tumor, as has been previously reported^24^,^25^. Voxel histogram analysis provides the ability to detect heterogeneous regions of signal enhancement (i.e. hotspots) as necessitated by molecular MRI^1^ (Fig. 3b). Hotspots were conservatively defined as those voxels within the top quartile of the histogram, isolating the extent of elongation of the right tail of the histogram due to C-SNAM nanoaggregation. A substantial elongation of the right tail of the voxel histogram of doxorubicin-treated mice administered C-SNAM (Fig. 3b, blue) can be seen relative to untreated mice administered C-SNAM (Fig. 3b, black) and treated mice administered the NC-ctrl (Fig. 3b, red) at 50 min post-contrast. Furthermore, the histograms shift to the left over time (50 min *versus* 110 min, Fig. 3b) to a substantially greater extent for treated mice receiving NC-ctrl relative to those receiving C-SNAM, suggesting a more rapid washout of the non-cyclized probe relative to the nanoaggregates. The change in tumor hotspot contrast over time was quantified as the percent increase of the mean hotspot signal relative to pre-contrast for a given time point (Fig. 3c). The signal enhancement was significantly greater for mice treated with metronomic chemotherapy and administered C-SNAM (Fig. 3c, blue) relative to both untreated mice (Fig. 3c, black) and treated mice administered NC-ctrl (Fig. 3c, red) (General linear model repeated measures analysis, *P* < 0.05, n = 4 mice per group). While the %SE from treated mice administered NC-ctrl increased initially relative to untreated mice administered C-SNAM, it rapidly decreased (within 70 min) to the levels of untreated mice, whereas C-SNAM maintained prolonged retention even beyond 2 hr. This rapid washout of NC-ctrl relative to C-SNAM is likely due to its inability to cyclize or self-assemble.

### Radiation Therapy Response Monitoring with C-SNAM

**Figure 4:**
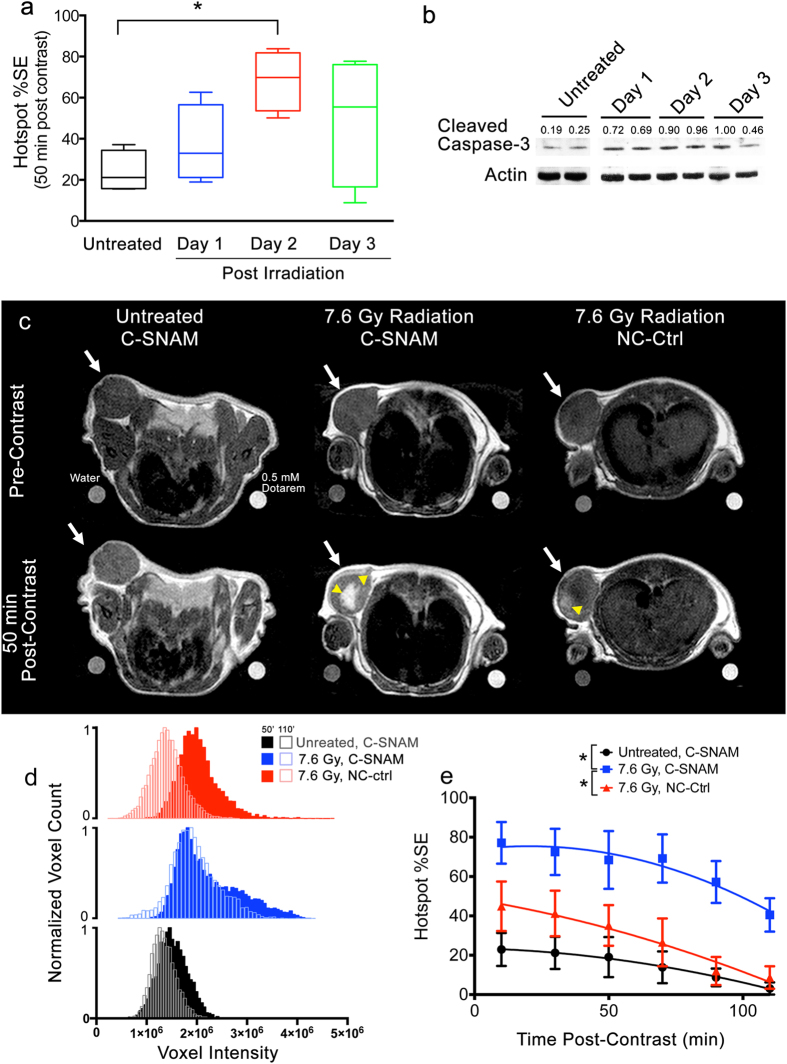
Imaging the therapeutic response to single dose radiation therapy in HeLa tumor-bearing mice. (**a**) Box plot showing hotspot %SE due to caspase-3 activation for untreated mice (black), or for mice receiving 7.6 Gy tumor irradiation and imaged 1 (blue), 2 (red), or 3 days (green) later. All imaging was performed with C-SNAM. *p < 0.05 (ANOVA), n = 4 mice per group. (**b**) Western blot following cleaved caspase-3 expression in untreated mice and over time after tumor irradiation. Values are cleaved caspase-3 band intensities normalized to actin loading control. (**c**) Representative MR images of untreated (left) and treated mice (center) receiving C-SNAM, and treated mice receiving NC-ctrl (right) pre-contrast (top) and 50 min post-contrast (bottom). Tumor is indicated by white arrow, and hot spots are indicated by yellow arrowhead. Water and 0.5 mM Dotarem phantoms are shown on bottom left and right, respectively, of each image. (**d**) Representative voxel histograms for untreated (black) and treated (blue) mice receiving C-SNAM, and treated mice receiving NC-ctrl (red). Histograms are shown 50 min post-contrast (solid bars) and 110 min post-contrast (open bars). (**e**) The percent signal enhancement (%SE) for hotspot signal enhancement (mean signal within the top quartile of the enhancement histogram) is plotted over time for untreated (black) and treated (blue) mice receiving C-SNAM, and treated mice receiving NC-ctrl (red). Values are mean ± s.d., *p < 0.05 (general linear model repeated measures), and n = 4 per group.

In order to determine the ideal imaging window for radiation treatment, mice were left untreated, or treated with a single 7.6 Gy dose of radiation from a 225 kV beam and imaged either 1, 2, or 3 days later (Fig. 4a,b). The maximum signal enhancement was observed 2 days following treatment (Fig. 4a) (ANOVA, *P* < 0.05, n = 4 mice per group). Tumor lysates were analyzed for cleaved caspase-3 expression, revealing that these levels increased from day 1 to day 2, and at day 3 began to decrease toward untreated levels (Fig. 4b), showing substantial variability at day 3 that corresponds well with the observed variation in imaging signal enhancement on that day (Fig. 4a, green). Therefore, the optimal imaging window for measuring caspase-3 activation of HeLa tumors under this experiment condition occurs 2 days following the radiation treatment.

With the determination of the imaging window, mice were imaged 2 days following a single administration of 7.6 Gy of radiation to tumors. Representative images of a tumor pre-contrast (Fig. 4c, top row) and 50 min post-contrast (Fig. 4c, bottom row) are provided for untreated mice (left) and mice treated with radiation and administered C-SNAM (middle), and treated mice receiving the NC-ctrl (right). Large hotspots (Fig. 4c, yellow arrowheads) of C-SNAM activation and retention can be observed in the treated mice, with fewer and less intense hotspots notable in untreated mice. While treated mice administered either C-SNAM or NC-ctrl probe exhibited average post-contrast signal enhancement similar to that of the untreated mice (Supplementary Fig. S2b), hotspot analysis revealed substantial differences. Voxel histogram analysis at 50 min post-contrast (Fig. 4d) revealed a substantial elongation of the right tail of the histogram for treated mice receiving C-SNAM (blue) relative to both untreated mice receiving C-SNAM (black) and treated mice receiving NC-ctrl (red). This elongation of the right tail of the histogram translated to a significant increase in the hotspot %SE for treated mice receiving C-SNAM (Fig. 4e, blue) relative to untreated mice (Fig. 4e, black) or treated mice receiving NC-ctrl (Fig. 4e, red) (General linear model repeated measures analysis, *P* < 0.05, n = 4 mice per group). Here, the rapid clearance of the unactivated probe in untreated mice and from mice administered the NC-ctrl is observed, whereas treated mice receiving C-SNAM display prolonged signal enhancement beyond 50 min post-contrast. This result is corroborated by the substantial left-shifting of the histograms of mice receiving NC-ctrl relative to those receiving C-SNAM between 50 min and 110 min (Fig. 4d), and may be attributed to the ability of the probe to be activated by caspase-3 and self-assemble *in situ* into nanoparticles.

As another control to investigate whether the caspase-3 dependent activation and self-assembly is required to enhance the hotspot %SE, a clinical small molecule contrast agent, ProHance®, which is unchanged by caspase-3, was applied to mice pre- and post-radiation treatment (Supplementary Fig. S3). Little observable increase in tumor signal enhancement can be observed for untreated *vs* radiation-treated mice (Supplementary Fig. S3a), and no significant difference in the tracer clearance kinetics from tumor tissue were detected between radiation-treated *vs* untreated mice (Supplementary Fig. S3b).

**Figure 5:**
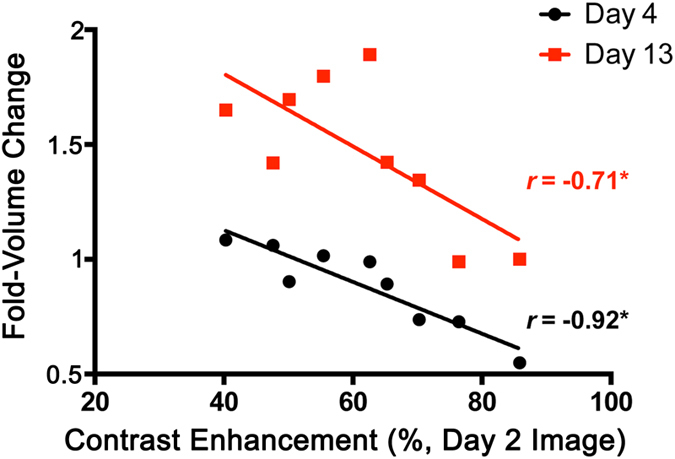
Interrogation of the relationship of C-SNAM MR signal enhancement with tumor volume change following radiation therapy. Tumor size change was measured as the fold-change in volume 4 days (black circles) or 13 days (red square) following the day of treatment administration (Fold-Volume Change). MR signal enhancement with C-SNAM was measured 2 days following tumor irradiation and was quantified as the hotspot %SE. Pearson’s correlation coefficients (*r*) are shown for day 4 and day 13 following treatment of the same group of animals. *p < 0.05 (Pearson’s *r*), n = 9.

Utilizing the optimal 2-day post-treatment time-point for a single imaging session of radiation-treated mice, the MR signal enhancement from C-SNAM was correlated with tumor treatment response (% tumor volume change) (Fig. 5). Tumor size was measured on the day of treatment administration, and then each day afterwards for 13 days (n = 9 mice). A significant correlation was observed between MR signal enhancement measured 2 days following radiation treatment and tumor volume change on both day 4 (black line, *r* = −0.92, Pearson correlation test, *P* < 0.05) and day 13 (red line, *r* = −0.71, Pearson correlation test, *P* < 0.05), representing early and late time points, respectively, in HeLa tumor growth. This negative correlation is expected since the higher the MRI signal produced by C-SNAM, the more caspase-3 activity, and the more tumor cell death and ensuing tumor growth reduction is expected. Therefore, the MR signal intensity from a single imaging session with C-SNAM within the optimal imaging window for therapy response monitoring is prognostic of the expected percent tumor volume change following therapy.

### Co-localization of C-SNAM and Caspase-3 *Ex Vivo* with MRI Signal Production *In Vivo*

**Figure 6:**
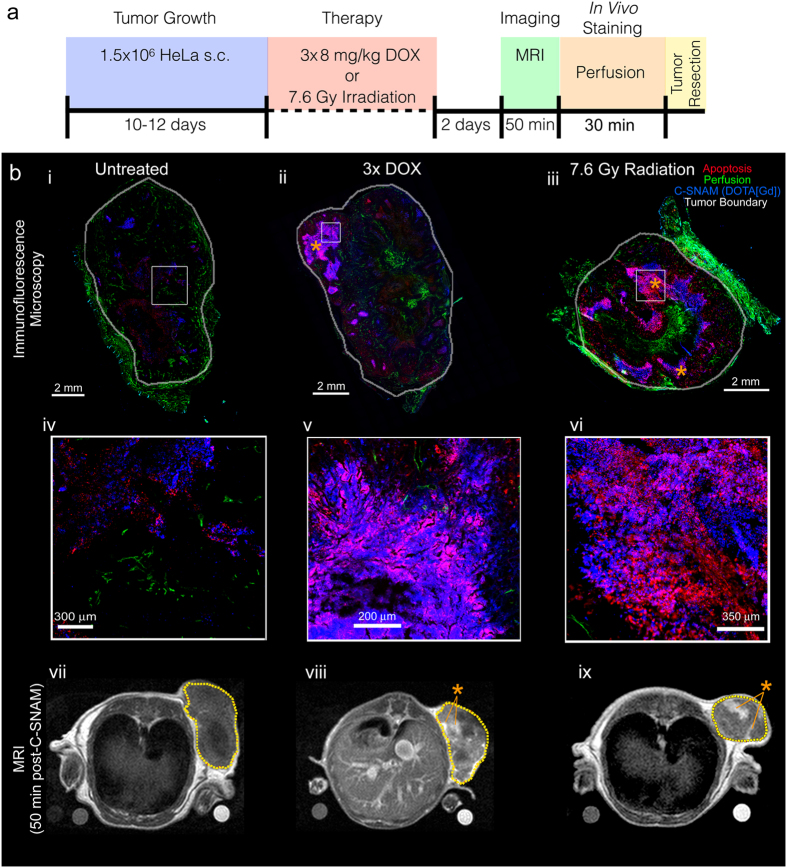
*Ex vivo* histological analysis of tumor perfusion, caspase-3 activation, and C-SNAM retention during tumor therapy, and relationship to *in vivo* molecular MR imaging. (**a**) Experimental design for histological analysis, where blue represents tumor growth phase, red represents treatment phase, green represents imaging phase, orange represents the *in vivo* staining of tumor perfusion, and yellow corresponds to animal euthanasia and tumor resection. (**b**) Whole section fluorescence microscopy (i–iii), enlarged regions of fluorescence microscopy indicated by white boxes (iv–vi), and MR images 50 min post-C-SNAM injection (vii–ix) approximately corresponding to tissue sections are displayed. Untreated mice (i, iv, vii), and mice treated with either metronomic chemotherapy (3x DOX—ii, v, viii) or single dose radiation therapy (7.6 Gy Radiation—iii, vi, ix) are shown. For immunofluorescence images, apoptosis as marked by active caspase-3 (red), tumor vascular perfusion as marked by isolectin binding (green), and C-SNAM as detected by anti-DOTA-metal chelate antibody (blue) are shown. *Ex vivo* tumors are marked by white line (i–iii) and tumor in MR images are marked by dotted yellow line (vii-ix). *indicates approximately aligned hotspots in MR images with regions of high caspase-3 activation and C-SNAM retention in immunofluorescence images.

In order to approximate the location of caspase-3 and C-SNAM with signal enhancement observed *in vivo*, untreated animals and animals receiving metronomic chemotherapy or single dose radiation therapy were subjected to imaging with C-SNAM 2 days following the termination of therapy (Fig. 6a). Thirty minutes prior to tumor resection, animals were administered DyLight™ 594-conjugated *Grifonia simplicifolia* isolectin B4 intravenously in order to label perfused blood vessels^26^, after which point tumors were resected for immunofluorescence staining. Representative stained tissue sections for untreated (Fig. 6b i), chemotherapy-treated (Fig. 6b ii), and irradiated animals (Fig. 6b iii) are shown, with white boxes indicating enlarged regions provided in Fig. 6b iv-vi, respectively. The MR images acquired 50 min post-contrast and corresponding to the vicinity and approximate orientation of the tissue sections are shown (Fig. 6b vii-ix, respectively). Regions of apoptosis, as indicated by caspase-3 expression (red) approximately match regions of signal enhancement in the MR images (mutually marked with*). Importantly, these apoptotic regions overlay with regions of C-SNAM retention (blue), as indicated by a recombinant anti-Gd-DOTA antibody previously described^27^,^28^. This overlay can be observed for apoptosis induced by both chemotherapy (Fig. 6b v) and radiation therapy (Fig. 6b vi), and as well for untreated tumors, representing the baseline homeostatic apoptosis that occurs throughout tumor progression^29^,^30^. In some regions of chemotherapy and radiation-treated tumors, there appears to be large amount of C-SNAM (blue) surrounded by apoptotic cells expressing caspase-3 (red) (Fig. 6b v, for example). This demonstrates one of the signal amplification mechanisms of C-SNAM: the ability of a single caspase-3 enzyme to activate a large amount of substrate-based C-SNAM monomers, resulting in large amounts of nanoaggregates in the immediate vicinity of apoptotic cells. Finally, perfusion can be observed throughout the tumor tissue (green), and not limited to regions of C-SNAM retention, confirming that MR signal enhancement is not a product of the vascular retention of C-SNAM, but rather corresponds to caspase-3-dependent probe retention in the apoptotic tumor regions.

## Discussion

As an enabling component of molecular MRI, activatable MRI probes have been designed for *in vivo* imaging of enzyme activity. One common strategy includes modulating the inner water coordination sphere of a metal chelator (i.e. ‘q’-activatable contrast agents) through reporter gene activity (e.g. β-galactosidase)^31^ or microenvironmental factors (e.g. pH, redox)^32^. However, with a single mechanism of signal amplification (thus potentially small signal enhancement) or for reporter gene applications, these probes have limited overall clinical relevance. Another strategy utilizes a myeloperoxidase-triggered oligomerization of contrast agent monomer for the detection of inflammation^33^,^34^,^35^. While myeloperoxidase is specific for inflammation, this strategy cannot be applied to image other enzyme targets^33^,^34^,^35^. When applied to monitoring certain cancer therapies, such as oncolytic virus treatment^34^, it suffers from artifacts due to the immune response similar to FDG, likewise preventing its application to radiation therapy monitoring^10^,^12^. The specificity of C-SNAM for apoptosis and not immune activity makes it demonstrably unique in its ability to detect radiation-induced apoptosis without high background levels.

As a marker for response to therapy, caspase-3 is an ideal target as it represents the earliest biochemical change that commits the cell to death^2^,^17^. While the use of caspase-3 as a marker of radiation response remains a controversial topic^29^,^36^,^37^, recent studies have reinforced the role of caspase-3 in eliciting radiation response^38^,^39^,^40^, which is supported by the strong correlation of C-SNAM activation and retention with the degree of radiation response (Fig. 5). Importantly, caspase-3 provides the opportunity for signal enhancement due to enzymatic processing of substrate-based probes. None of the other targets pursued for the molecular imaging of tumor response to therapy, such as phosphatidyl serine flipping^22^,^41^,^42^, mitochondrial membrane potential^43^, plasma membrane depolarization^44^, and DNA fragmentation^45^, are enzymes^3^, and as a result lack the signal amplification that is key for successful molecular MR imaging^1^. Importantly, other caspase-3-targeted imaging agents with clinical relevance (e.g. ^18^F-ICMT-11^24^) are activity-based probes that bind the enzyme and inhibit its activity. An inhibitor based MR probe would interfere with the induced treatment effect^3^, since it abrogates the benefit of signal amplification afforded by the enzymatic activity and likely needs a high imaging dose.

C-SNAM provides a unique strategy to amplify signal enhancement through *in situ* nanoaggregation, triggered by the activation of caspase-3 and death of tumor cells. The chemistry of C-SNAM and the activation mechanism has been comprehensively characterized *in vitro* to demonstrate enhanced C-SNAM uptake and retention in apoptotic cells^14^. The combination of a substrate-based probe, the formation of a nanoparticle to provide enhanced MR signal production through an increase in *r*_1_ (Fig. 1c), and prolonged tissue retention of activated product (Figs 3 and 4) makes this unique approach to molecular MR imaging successful *in vivo*. Additionally, apoptosis eventually compromises the integrity of the cell membrane^46^, allowing for the effective penetration of C-SNAM^14^ into apoptotic cells, with prolonged retention of the nanoaggregates as we have observed with the fluorescent analog of C-SNAM with superresolution microscopy^15^.

The administered dose of C-SNAM was 0.1 mmol/kg for all our imaging experiments. Using the calculation of human equivalent dose described by the FDA^47^, the corresponding clinical dose of C-SNAM for tumor treatment response monitoring would be 0.008 mmol/kg, more than an order of magnitude below the dose prescribed for gadolinium-containing agents in humans (0.1 mmol/kg)^48^. This low required dose can be attributed to the caspase-3-triggered *in situ* nanoaggregation and MR signal amplification strategies for identifying tumor death, since neither the non-cyclizable control (NC-ctrl, Figs 3 and 4) nor a small molecule MRI contrast agent ProHance® (Supplementary Fig. S3) was able to identify the response to therapy.

The treatment models employed in this work produced moderate treatment response, only reducing tumor growth rates relative to untreated tumors but not dramatically changing tumor size (Fig. 2b). Even with these moderate treatment effects, a single, post-treatment imaging study was sufficient to determine the efficacy of chemotherapy (Fig. 3) or radiation therapy (Fig. 4). The intensity of the imaging signal was found to directly correlate to the long-term treatment outcome (Fig. 5), suggesting that C-SNAM can detect molecular-level changes in tumors prior to the change in tumor volume. The levels of active caspase-3 were directly compared in radiation or doxorubicin-treated tumors (Fig. 2c), showing that radiation treatment results in an increased level of active caspase-3 than 3X DOX (semi-quantitative western blot band intensities or 0.84 and 1 after radiation, versus 0.62 and 0.93 after chemotherapy). This increase in apoptosis following radiation therapy relative to chemotherapy was also observed through immunofluorescence staining (Fig. 6). Importantly, this data is consistent with the MR images generated with C-SNAM administration that show an increased signal enhancement (Figs 3 and 4) following radiation treatment, suggesting that the focused delivery of radiation to HeLa tumors is more therapeutically effective than systemic chemotherapy. With this molecular-level therapy response data provided by C-SNAM, cancer treatments could be refined by adjusting applied dose, or by discontinuing ineffective therapy and initiation of alternate treatment options, for example the cessation of ineffective systemic chemotherapy and initiation of radiation therapy.

Enzyme-triggered nanoaggregation harnesses the benefits of enhanced retention and MR signal generation typical of nanoparticles, but utilizes small molecule probes and so overcomes the safety concerns generally limiting the use of nanoparticles in clinical imaging^49^. To further improve upon the tolerability of this new MR probe, particularly concerning the tissue residence lifetime of formed nano-aggregates, the probe scaffold can be made to self-immolate and induce nanoparticle disassembly after imaging is completed (approximately 2 to 4 hours), as has been shown with other nanoparticles^50^,^51^. This would ensure that the probe enters and leaves the body as a small molecule, allowing efficient clearance of the metal chelate, which merely resides in apoptotic tumor tissue temporarily. Another potential challenge in imaging tumor response to therapy is the dynamic nature of caspase-3 activity^18^. Caspase-3 is activated some time after the administration of radiation or chemotherapy when one or more signaling pathways eventually turn on caspase-3 and commit the cell to die^17^. Once activated, caspase-3 initiates cell death, including the degradation of biomolecules and packaging of the cell into apoptotic bodies in preparation for removal from local tissue. Imaging too early, prior to its activation, or too late, beyond the clearance of the cell from local tissue, would miss the effects of this enzyme in response to applied therapy^18^. C-SNAM was capable of addressing this challenge by determining the imaging window for optimal response monitoring (Fig. 4a), a method that can be applied clinically through multiple sequential imaging sessions free from repeated exposure to ionizing radiation and at low contrast agent doses. While the variability of this imaging window would need to be assessed across tumor types and treatment options, C-SNAM now provides a means by which this important clinical question can be addressed.

In summary, we have shown that our approach to activatable molecular MRI using C-SNAM is applicable to monitoring a range of therapeutic strategies, including radiation therapy that has previously been elusive for clinical imaging agents^10^,^12^. The development and implementation of C-SNAM realizes a technical advance towards the transformative paradigm of molecular medicine: the ability to monitor subcellular effects of applied therapy prior to tumor size change, but predictive of long-term outcome, in order to inform the application of personalized cancer treatment.

## Materials and Methods

### Chemicals and Materials

All chemicals and materials were purchased from indicated commercial sources. C-SNAM and NC-ctrl were synthesized and characterized as previously described^14^. Scanning electron microscopy was obtained using a solution of C-SNAM (100 μM, 500 μL) in caspase buffer (50 mM HEPES, 100 mM NaCl, 1 mM EDTA, 10 mM tris(2-carboxyethyl) phosphine hydrochloride, 10% glycerol and 0.1% 3-((3-cholamidopropyl)dimethylammonio)-1-propanesulfonate, pH 7.4), incubated with or without human recombinant caspase-3 (50 nM) at 37 °C for 6 h. Then, 3 μL of solution was dropped onto a clean silicon chip (SPI Supplies^®^/Structure Probe Inc., West Chester, PA) that was mounted on the imaging stub using double-sided conductive tape. The samples were put into a dust-free container, and allowed to dry in the air for 3 days. The nanoaggregates were examined using a Nova NanoSEM (FEI, Hillsboro, OR) scanning electron microscope operating at an accelerating voltage of 3 Kv.

### Relaxivity Measurements

A series of solutions containing contrast agents with four to five different concentrations (0.05–0.5 mM) in caspase buffer were treated with or without caspase-3 (50 nM) at 37 °C overnight. The solutions were then placed in 100 μL eppendorf tubes and imaged at 1T (Bruker Icon, Bruker) at room temperature. The scanning procedure began with a localizer and then consisted of a series of inversion-prepared fast spin-echo scans, identical in all aspects (TR 6000 ms, TE minimum, field of view 6 cm, slice thickness 2 mm, matrix 128 × 128, NEX 1) except for the inversion time (TI) which was varied as follows: 4000, 2400, 1200, 800, 600, 400, 300, 200, 100, and 50 ms. This scanning procedure produced enough data to fit quantitative T_1_ relaxation values for each sample in the image. For quantitative data analysis, signal intensities were extracted from each of the 5 samples at each of the 10 TI times by manual region of interest (ROI) placement and voxel averaging within the ROI. Signal intensity versus TI relationships were fit to the following exponential T1 recovery model by non-linear least squares regression: SI (TI) = S0 [1–2*exp(−TI/T1) + exp(−TR/T1)]. Relaxation rates (R1) were determined as 1/T1. Longitudinal relaxivities (*r*_1_, units of mM^−1^ s^−1^) were calculated as the slope of R1 *vs.* [Gd] after determination of true Gd concentration for each sample using inductively coupled plasma-mass spectrometry (ICP-MS) measurement.

### Cell Culture

HeLa human cervical carcinoma cells were grown in Dubelco’s Modified Eagle’s Medium (DMEM) (Life Technologies, Inc., Carlsbad, CA) supplemented with 10% fetal bovine serum and 1% PenStrep (100 U/mL penicillin, 100 μg/mL streptomycin, Life Technologies, Inc.). Cells were certified pathogen-free by PCR prior to implantation into animals.

### Animal Models

Animal care and euthanasia were performed in accordance with protocols approved by the Administrative Panels on Laboratory Animal Care of Stanford University. Six-week old female nude (nu/nu) mice (Charles River Laboratories International, Inc. Wilmington, MA), were inoculated with 1.5 × 10^6^ HeLa cells suspended in 50 μL of 50% v/v Matrigel:DMEM *subcutaneously* in the right shoulder. When tumors reached 0.7 cm in any aspect (approx.10–12 days), tumor treatment was initiated. For metronomic chemotherapy, 8 mg/kg doxorubicin was administered *intravenously* every 4 days for a total of three rounds of chemotherapy. Radiation therapy was delivered with a single 225 kV beam using the Kimtron IC 225 irradiator (Kimtron Medical, Woodbury, CT). Mice were anesthetized by ketamine/xylazine (80 mg/kg ketamine and 5 mg/kg xylazine) and were affixed to a lead shield containing perforations through which tumors were exposed. This way, only the subcutaneous tumors received a dose of 7.6 Gy, which was measured *in vivo* with thermoluminiscent dosimeters calibrated in the treatment beam. For all mice, total mouse body weight and tumor size (width and length by caliper measurement) was recorded every other day. Tumor volumes were calculated assuming ellipsoid shape according to (length × width^2^)/2. For all imaging studies, n = 4 mice were used per treatment group. For the correlation of imaging signal with therapeutic outcome, n = 9 mice were used.

### *In Vivo* Fluorescence Imaging

Two days following the final treatment, 5 nmol of quenched fluorescent probe, as previously described^15^ (Supplementary Fig. S1), in saline was injected *i.v.* and fluorescence imaging was performed 4 hrs later on an IVIS Spectrum (PerkinElmer Inc., Waltham, MA) using a 675 ± 25 nm excitation filter and 720 ± 10 nm emission filter. Regions of interest (ROIs) were drawn over the tumor and the thigh of the animal in order to calculate the tumor-to-leg intensity.

### *In Vivo* MRI & Data Analysis

All images were acquired on a Bruker ICON (Bruker, Billerica, MA) 1 Tesla small animal MRI system using the commercial 50 mm mouse body radio frequency coil. Paravision software was used to implement a rapid acquisition with relaxation enhancement (RARE) pulse sequence (TR = 446 ms, TE = 15 ms, ETL = 2, Averages = 16) providing 150 μm in-plane resolution and 1 mm slice thickness. Animals were imaged under isoflurane anesthesia prior to contrast agent injection. Contrast agent, either 0.1 mmol/kg C-SNAM, 0.1 mmol/kg NC-ctrl, or 0.2 mmol/kg ProHance (R), was injected through a venous microport (Braintree Scientific, Inc., Braintree, MA) to prevent movement of animals, and the microport was then flushed with saline and images were acquired every 10 min for 120 min. Images were acquired either 24, 48, or 72 hours post-radiation.

Images were analyzed using Inveon Research Workplace (Siemens Medical Solutions USA, Inc., Malvern, PA). Pre-contrast, and post-contrast images (10, 30, 50, 70, 90, and 110 min) were analyzed. ROIs were drawn around tumors, slice-by-slice, for the volume of the tumor, and voxel intensities were plotted as histograms in order to identify the value of the 75th percentile. The percent difference in the intensity value of the 75^th^ percentile was calculated for post-contrast relative to pre-contrast images, yielding the 75%ile percent signal enhancement (%SE) values.

### Expression and Purification of Anti-DOTA Antibody

A custom mouse-derived FLAG-tagged antibody for DOTA-metal chelates, as previously reported^27^,^28^,was utilized to visualize C-SNAM localization *ex vivo*. The Gwiz plasmids containing Sm3e/C825 light chain insert and Sm3e heavy chain insert for antibody expression were kindly provided by Prof. K.D. Wittrup (Massachusetts Institute of Technology, USA). Small-scale purification was carried out as previously described^52^. Briefly, 80 μg of each plasmid along with 1 mg/mL of polyethyleneimine (molecular weight of 25000) at pH 7.0 were transiently transfected into 160 mL (1 × 10^6^ cells/mL) of FreeStyle^TM^ 293-F cells (Life Technologies) grown in FreeStyle™ 293 expression medium (Life Technologies) at 37 °C, 5% CO_2_. The supernatant was collected 6–8 days after transfection. Overexpressed antibody was purified using Protein A agarose resin (Pierce Biotechnology, Inc., Rockford, IL) according to the manufacturer’s protocol. Purity and size of the final product was confirmed by 10% SDS-PAGE (Supplementary Fig. S4) with obtained purity comparable to previous reports^52^.

### Histological Analysis

Animals were inoculated with HeLa tumors and treated as previously described. DyLight 594-labelled *Griffonia simplicifolia* isolectin B4 (Vector Labs, Inc., Burlingame, CA) was dissolved in 1 mM CaCl_2_ in saline to 0.5 mg/mL^53^. Immediately following MR imaging, 100 μL of this isolectin solution was injected *i.v.*, and mice were euthanized 30 min later by cervical dislocation under deep anesthesia. Tumors were immediately resected, photographed so that rostral-caudal and medial-lateral axes were known, fixed overnight in 10% buffered formalin and then in 30% sucrose solution. Tumors were then embedded in optimal cutting temperature medium so that section occurred from the caudal end of the tumor in an axial plane approximating that displayed in the MR images, and sectioned axially in 14 μm sections. Sections were obtained every 100 μm so that sections could be approximately matched to MR images representing 1 mm tumor sections, and those sections corresponding to tumor locations showing signal enhancement were processed for immunofluorescence imaging. Sections were stained with rabbit-derived cleaved caspase-3 (Asp175) primary antibody (9661, Cell Signaling Technology, Inc., Danvers, MA) and Alexa 647-goat-anti-rabbit IgG secondary antibody (Life Technologies, Carlsbad, CA) to detect apoptosis, as well as the custom mouse-derived FLAG-tagged primary antibody for DOTA-metal chelates, which was visualized utilizing an Alexa 405-anti-FLAG secondary antibody (Life Technologies, Inc.). Incubations with primary antibodies were performed overnight at 4 °C, while secondary antibodies were incubated 1 hr at room temperature in the dark. Slides were sealed and imaged using a Zeiss AxioImager M1 upright widefield fluorescence microscope (Carl Zeiss AG, Ltd., Thornwood, NY) with Sony ICX 285 charge-coupled device camera (Sony Corp., New York, NY). Images were acquired utilizing 365 ± 50 nm/445 ± 50 nm (Alexa 405), 560 ± 40 nm/630 ± 75 nm (Alexa 594), and 640 ± 30 nm/690 ± 50 nm (Alexa 647) filter sets on 10x objective (Plan Apochromat, NA = 0.45) with automated tiling (10% overlap) over the entire tumor section area. Shortest exposure times across all samples were selected for each fluorescent channel and all images were acquired at this setting to enable comparison. Tiling was performed using Zen software (Carl Zeiss AG, Ltd.) and images were processed (scale bar, zoomed sections) using ImageJ-64 bit^54^.

### Western Blot

Animals were inoculated with HeLa tumors and treated as previously described above. At indicated time points, animals were euthanized by CO_2_ inhalation followed by cervical dislocation. Tumors were immediately homogenized utilizing a PowerGen 125 hand-held homogenizer (Thermo Fisher Scientific, Waltham, MA) in ice-cold RIPA buffer at 500 μL/10 mg tissue (Sigma-Aldrich, St. Louis, MO) containing HALT™ protease inhibitor cocktail (Thermo Fisher Scientific) and prepared for loading into 10% poly(acrylamide) gel as recommended by the manufacturer. Protein concentrations were determined by Bradford Assay (Bio-Rad Laboratories, Inc., Hercules, CA) as recommended by the manufacturer, and 50 μg protein was loaded into each lane. Proteins were transferred to 0.45 μm pore-size poly(vinylidene fluoride) membranes, which were blotted with rabbit-derived cleaved caspase-3 (Asp175) antibody (Cell Signaling Technology, Inc.). Bands were visualized by incubation with horseradish peroxidase-conjugated goat anti-rabbit secondary antibody (ab6721, Abcam, Cambridge, UK) and ECL western blotting substrate (Thermo Fisher Scientific). Membranes were stripped and reprobed for β–actin (PA1-46296, Thermo Fisher Scientific) as loading control. Band intensities were calculated using ImageJ-64 bit^54^, and represented as intensity of cleaved caspase-3 normalized to actin intensity.

### Statistical Analyses

Results are expressed as the mean ± standard deviation unless otherwise stated. Statistical comparisons between two groups were determined by one-way ANOVA followed by a post-hoc Tukey’s HSD test. Time course analysis between groups was performed by general linear model repeated-measures analysis. Correlation analyses were performed by one-tailed Pearson’s *r*. Data sets were assessed for normality and equal variance prior to statistical evaluation. For all tests, *p* < 0.05 was considered statistically significant. All statistical calculations were performed using GraphPad Prism v. 5 (GraphPad Software Inc., CA), except for general linear model analyses, which were performed using SPSS (IBM).

## Additional Information

**How to cite this article**: Shuhendler, A. J. *et al.* Molecular Magnetic Resonance Imaging of Tumor Response to Therapy. *Sci. Rep.* **5**, 14759; doi: 10.1038/srep14759 (2015).

## Supplementary Material

Supplementary Information
