# Supplementary material for: Molecular Magnetic Resonance Imaging of Tumor Response to Therapy
Source: Sci Rep. 2015 Oct 6;5:14759. doi: 10.1038/srep14759 (PMC4594000; doi:10.1038/srep14759)
Supplement: Supplementary Information [file srep14759-s1.pdf]

Supplementary Information

Molecular Magnetic Resonance Imaging of Tumor Response to Therapy

Adam J. Shuhendler [1,2‡], Deju Ye [1,2‡], Kimberly D. Brewer [1,2‡], Magdalena Bazalova-Carter [1,3], Kyung-Hyun Lee [1,2], Paul Kempen [4], K. Dane Wittrup [5], Edward E. Graves [1,3], Brian Rutt [1,2*], Jianghong Rao [1,2*]

[1]Molecular Imaging Program at Stanford, Departments of [2]Radiology, [3]Radiation Oncology, and [4]Materials Science and Engineering, Stanford University, Stanford, California 94305, USA
[5]Department of Chemical Engineering, Department of Biological Engineering, and Koch Institute for Integrative Cancer Research, Massachusetts Institute of Technology, Cambridge, Massachusetts, 02139, USA

E-mail: brutt@stanford.edu; jrao@stanford.edu

[‡]Corresponding authors.

*These authors contributed equally to the work.

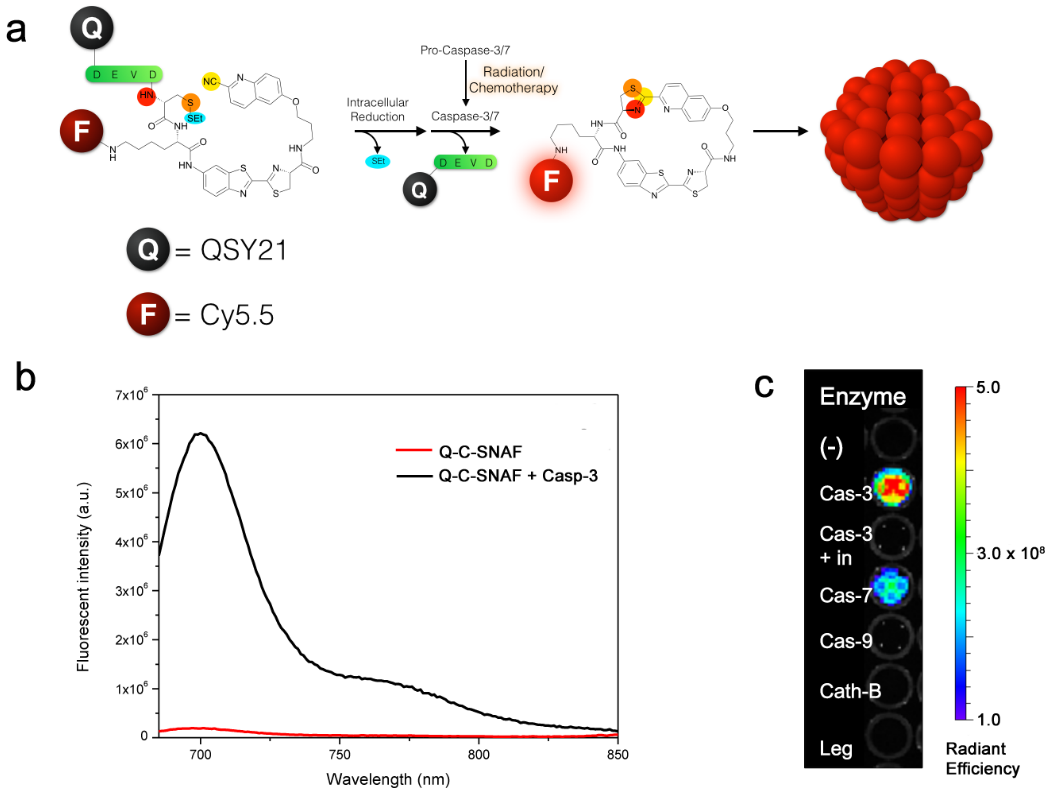

Supplementary Figure S1. Structure, mechanism of activation, and *in vitro* characterization of Q-C-SNAF, a quenched fluorescent analog of C-SNAM. (**a**) The structure of Q-C-SNAF is shown with a fluorophore (Cy5.5, red sphere) linked to the cyclizable backbone of the probe, and a quencher (QSY21, black sphere) linked to the terminal aspartate of the DEVD capping peptide. Upon cleavage of DEVD by caspase-3, the capping group and the quencher are removed, restoring Cy5.5 fluorescence and inducing backbone cylclization, ultimately resulting in self-assembly to fluorescent nanoparticles. (**b**) Fluorescence emission scans of Q-C-SNAF alone (red) or after incubation with active caspase-3 (black). Near infrared fluorescence can be seen after caspase-3 activation due to removal of the quenching group and fluorescence emission of Cy5.5. (**c**) Imaging of the specificity of activation of Q-C-SNAF with various caspases (caspase-3, -7, -9), cathepsin B, and legumain proteases. Additionally, caspase-3 was inhibited by the commercial inhibitor Z-VAD-fmk (Sigma-Aldrich Co., St. Louis, MO), preventing activation of Q-C-SNAF (cas-3+in).

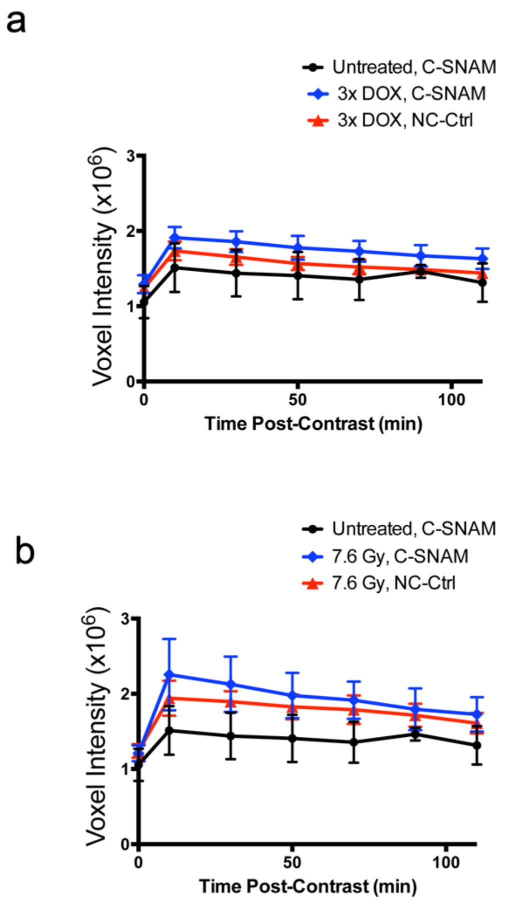

Supplementary Figure S2. Plots of the mean change in tumor intensity over time post-contrast for mice receiving metronomic chemotherapy (a) or radiation treatment (b). In each treatment arm, mice were either left untreated (black) or treated (blue) and administered C-SNAM for imaging, or were treated and administered NC-ctrl (red). Values are mean±s.d. for n=4 mice.

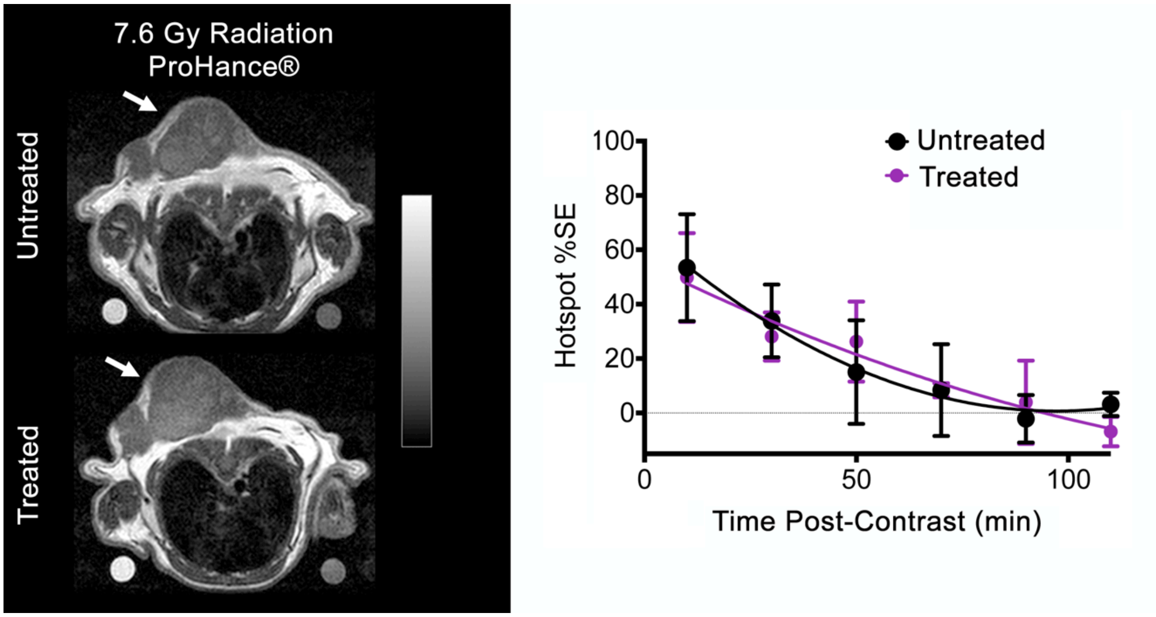

Supplementary Figure S3. Small molecule contrast agents (ProHance®) applied at clinically-relevant doses show no difference in tumor contrast enhancement following radiation therapy. (**a**) Representative MR images of untreated (top, pre-treatment) and treated mice (bottom, post-treatment) receiving ProHance®. Images shown are 50 min post-contrast injection. Tumor is indicated by white arrow. Water and 0.5 mM Dotarem phantoms are shown on bottom right and left, respectively, of each image. (**b**) The percent signal enhancement (%SE) for the hotspot (mean signal within the top quartile of the histogram) is plotted over time for untreated mice (black) or treated mice (purple) following single 7.6 Gy irradiation of tumors.

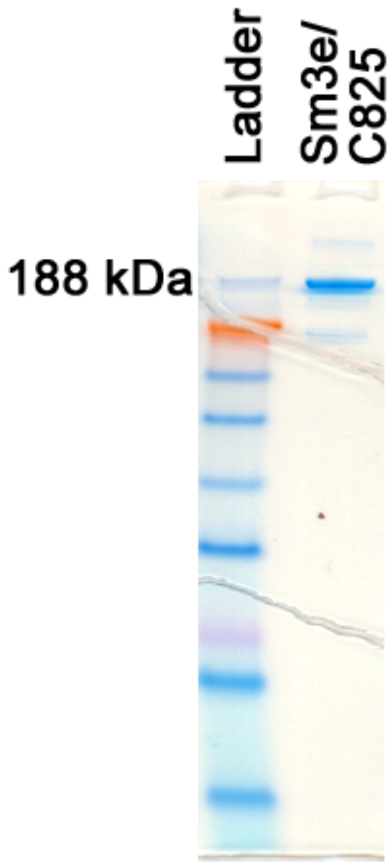

Supplementary Figure S4. Poly(acrylamide) gel electrophoresis of purified anti-DOTA-metal chelate antibody (Sm3e/C825). Purity achieved is similar to that previously reported[1].

References

1. Orcutt, K. D. et al. A modular IgG-scFv bispecific antibody topology. Protein Eng Des Sel. 2010;**23**:221-228.
